# The Effect of Telemedicine in Glycemic Control in Adult Patients with Diabetes during the COVID-19 Era—A Systematic Review

**DOI:** 10.3390/jcm12175673

**Published:** 2023-08-31

**Authors:** Fiorella Sotomayor, Reynier Hernandez, Rana Malek, Nehu Parimi, Elias K. Spanakis

**Affiliations:** 1 Division of Endocrinology, Diabetes, and Nutrition, University of Maryland School of Medicine, Baltimore, MD 21201, USA; fiorellasv89@gmail.com (F.S.); reynier.hernandez@som.umaryland.edu (R.H.); rmalek@som.umaryland.edu (R.M.); 2 Division of Endocrinology, Baltimore Veterans Affairs Medical Center, Baltimore, MD 21201, USA; josephtheressa.parimi@va.gov

**Keywords:** COVID-19, diabetes, glycemic control, pandemic, telemedicine

## Abstract

Telemedicine can be an effective tool for managing chronic diseases. The disruption in traditional diabetes care resulting from the COVID-19 pandemic led to global interest in telemedicine. With this manuscript, we evaluated the use of telemedicine for the management of diabetes during the pandemic and its impact on glycemic control, focusing on retrospective and prospective studies which included adult, non-pregnant patients with diabetes. We evaluated whether there was an improvement in HbA1c, time in range (TIR), glucose management indicator (GMI), mean glucose values, hypoglycemic episodes, time below range (TBR), or hospitalizations for hypoglycemia/DKA, depending on the available information provided. This review article highlights the benefits of telemedicine during the global state of emergency, which altered the standard of healthcare delivery. Across the studies reported in this review, telemedicine was shown to be an effective tool for the management of diabetes, illustrating its potential to be the new standard of care. Although these improvements may be confounded by potential extraneous factors present during the pandemic, telemedicine was shown to positively impact glycemic control. Overall, this article highlights the benefits of telemedicine on glycemic control during the global state of emergency, which altered the standard of care. With the rollback of COVID-19 restrictions, and a return to the office, this article emphasizes the necessity to study how telemedicine can be best utilized for diabetes management when compared to the traditional standard of care.

## 1. Introduction

Diabetes is one of the most prevalent diseases worldwide. According to the CDC, from 2001 to 2020, the prevalence of diabetes significantly increased among adults in the United States. Furthermore, the CDC estimated that 37.3 million people, representing 11.3% of the US population, have diabetes [1]. Notably, the global prevalence is expected to rise to 578 million by 2030 [2]. Following COVID-19 pandemic declaration, patients with diabetes were found to be at particularly high risk of intensive care admission (ICU) and mortality from COVID-19 infection, representing a vulnerable population [3,4,5,6]. The advent of the pandemic ushered in a new era in medical care, especially for diabetes, by allowing telehealth to become a key alternative tool that can help modernize care through the use of tools such as continuous glucose monitors, smart pens, and smart phones [7]. The outbreak of the COVID-19 pandemic created an additional challenge in providing care for chronic diseases such as diabetes. Given its highly contagious nature and propensity to spread from one person to another through direct transmission, measures such as social distancing, lockdowns, and travel restrictions were implemented to mitigate virus spread and reduce hospitalizations in different parts of the world, which led countries to adapt different strategies [8]. In the United States, there was a significant drop in in-person outpatient visits, prompting a shift towards the use of telemedicine as a consequence [9]. However, the impact of the pandemic extended beyond the United States and had a major repercussion in care across different countries around the world and medical specialties [10]. 

Overall, the change in the landscape of medical care posed a challenge to the way healthcare was delivered. Consequently, institutions increasingly utilized virtual clinics and telemedicine interventions to provide appropriate care for patients, including those with diabetes, to protect against COVID-19 infections. Despite the sudden change in care, telemedicine was positively received by patients [11,12]. Telemedicine is defined by the Institute of Medicine as “the use of electronic information and communications technologies to provide and support health care when distance separates the participants” [13]. The Centers for Medicare and Medicaid Services (CMS) describes telemedicine as “the exchange of medical information from one site to another through electronic communication to improve a patient’s health” [14]. Telemedicine can be an effective tool for more than just patients with an established diabetes diagnosis. It can also be used to navigate challenging situations such as insulin pump training through virtual clinics or management of new-onset diabetes, circumstances where in-person care were traditionally deemed necessary [15,16]. Although telemedicine was not broadly used prior to the onset of the pandemic, it swiftly became an instrumental tool for the care of patients with diabetes; that, in conjunction with the use of technology such as continuous glucose monitors (CGM), allowed physicians to provide adequate care and makes telemedicine feasible [17]. 

The COVID-19 pandemic led to worldwide interest in telemedicine, as evidenced by the multiple publications presented in this paper. In this article, we evaluate the use of telemedicine for the management of diabetes by presenting a comprehensive review of papers that focused on the use of telemedicine on glycemic control in adults after the COVID-19 pandemic declaration. 

## 2. Methods

**Figure 1:**
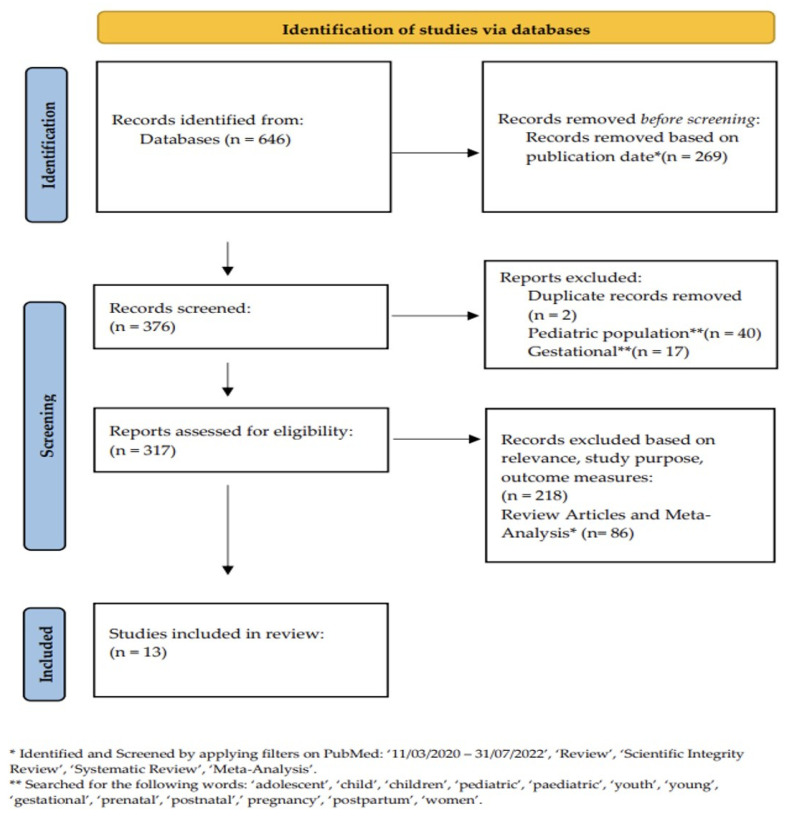
Database Search.

An electronic search of PubMed was conducted by two independent reviewers (F.S., R.H.) to analyze publications relating to diabetes management, telemedicine, and COVID-19. The search was conducted via PubMed advanced search builder using the following key words: ‘Diabetes telemedicine clinic and COVID-19′, or ‘Glycemic control telemedicine clinic and COVID-19′, or ‘Diabetes management and SARS-CoV lockdown’, or ‘Telemedicine diabetes and lockdown’ or ‘Impact telemedicine and diabetes control lockdown’. The search resulted in a total of 646 articles, which we filtered based on publication date. Using ‘11 March 2020–31 July 2022′, a total of 376 records remained. Two duplicate records were removed, and those that included pediatric patients or pregnant patients were excluded. From the 317 reports that remained, a filter was used to exclude review articles, systematic reviews, and meta-analysis articles. The remaining articles were screened for relevance, study purpose, and outcome measures. Those that did not have glycemic control evaluation as either primary or secondary end points, did not describe the impact of telemedicine on diabetes management during the pandemic, or studied diabetes comorbidities were excluded (Figure 1). This review did not focus on the financial impact of telemedicine. 

In the included studies, time in range (TIR), time above range (TAR), glucose management indicator (GMI), mean glucose value, postprandial plasma glucose (PPPG), fasting plasma glucose (FPG), hemoglobin A1c (HbA1c), time below range (TBR), and hypoglycemic events were used as parameters for evaluating glycemic control. Glucose monitoring methods used to monitor patients included continuous glucose monitor (CGM), self-monitoring of blood glucose (SMBG), and flash glucose monitoring (FGM). Additionally, multiple daily insulin injections (MDI), continuous subcutaneous insulin infusion (CSII), and non-insulin hypoglycemic medications (oral hypoglycemic agents and GLP-1 agonists) were among the different glucose treatment methods used in the various studies. (Table 1 and Table 2). 

## 3. Results

### 3.1. Evidence from Retrospective Studies

Among the retrospective studies published (Table 1), three of them assessed patients exclusively with type 1 diabetes (T1D) and included patients who used insulin pumps or MDI as methods of treatment and either CGM or FGM as glycemic monitoring methods [18,19,20].

A study conducted with 30 T1D patients on hybrid closed loop (HCL) insulin pumps [18] evaluated glycemic control through telemedicine across four different time points during the pandemic lockdown period (two weeks before the lockdown, Time 0), during the first two weeks of lockdown (Time 1), last two weeks of lockdown (Time 2), and first two weeks after the lockdown (Time 3) [18]. The study found an improvement in mean glucose value (155 mg/dL in Time 0 vs. 153 mg/dL in Time 3, *p* = 0.004), a significant improvement in TIR (68.5% in Time 0 vs. 73.5% in Time 3, *p* = 0.012) without an increase in level 1 (54–69 mg/dL) and level 2 (<54 mg/dL) hypoglycemia. The improvement in TIR was instead associated with a reduction in TAR (Table 1).

Another study by Boscari et al. [19], which enrolled 71 T1D patients managed by either MDI or CSII, analyzed the efficacy of telemedicine by comparing CGM/FGM combined data gathered four weeks before and four weeks after patients attended a telephone visit. This study showed a reduction in GMI from 7.16 to 7.05%, *p* = 0.002, a reduction in mean glucose value from 161.1 mg/dL to 156.3 mg/dL, *p* = 0.001, a reduction in TAR (>180 mg/dL) from 33.4 to 30.5%, *p* = 0.002, with an improvement in TIR (70–180 mg/dL) from 63.6 to 66.4%, *p* < 0.001. Furthermore, among those managed by CSII, there was a reduction in mean glucose value from 157.9 mg/dL to 152.6 mg/dL, *p* = 0.003 [19]. No changes were observed in TBR (<70 mg/dL) with 3.0 vs. 3.2% *p* = 0.6, respectively.

Alharthi et al. [20] evaluated patients with T1D and compared glycemic control from FGM device data in a total of 101 patients who attended a specialized diabetes clinic during the six-week lockdown period 61 patients attended a telemedicine visit (TM) and a total of 40 patients did not [20]. The study showed improvements in average blood glucose from 180 mg/dL to 159 mg/dL, *p* < 0.01 in those who attended a TM visit vs. 159.5 to 160 mg/dL *p* = 0.99 in those who did not. An improvement in TIR (70–180 mg/dL) from 46.0% to 55.0%, *p* < 0.01 vs. 58.0 to 57.0%, *p* = 0.20, was also observed. The authors also found a reduction in GMI from 7.7 to 7.2%, *p* = 0.03 vs. 7.3 to 7.2%, *p* = 0.65 in those who attended a TM visit vs. those who did not attend a TM visit, respectively. Additionally, a reduction in TAR (>180 mg/dL) was noted, without any significant change in TBR (<70 mg/dL) or in hypoglycemic events [20].

Four studies explored the impact of telemedicine on glycemic control in patients with type 2 diabetes (T2D) [21,22,23,24]. These studies monitored glycemic control through SMBG, fasting, or postprandial blood glucose. Unlike the studies mentioned above, none of the subjects used a continuous or flash glucose monitor. In addition, a wide range of medications, such as insulin, GLP-RA, and SGLT2i, were used for glucose control in these studies; insulin pumps in patients with type 2 diabetes were not explored.

Scoccimarro et al. [21] evaluated 269 patients and assessed the difference in HbA1c and body weight between the pre-lockdown and post-lockdown periods (from November 2019 to February 2020 vs. May to June 2020). They found no deterioration in metabolic profile but rather a slight improvement in HbA1cHbA1c% (7.3% ± 3.1% pre-lockdown vs. 7.2% ± 3.2% post-lockdown, *p* < 0.01) and in weight (83.2 ± 16.8 kg vs. 81.6 ± 16.4, *p* < 0.01) in the entire cohort.

In another study, Dutta et al. [22] compared glycemic control among a cohort of 96 patients with T2D who were followed for a six-month period through telemedicine or in-person visits [20]. The study found a reduction in HbA1c from baseline 8.7% ± 1.8 to 6.9 ± 1.1 in the telemedicine compared to the in-person group, which had a reduction in HbA1c from baseline 8.6% ± 2.1% to 7.0% ± 1.0%, *p* = 0.88 at six months follow-up. A reduction in FPG (fasting plasma glucose) and PPPG (post prandial plasma glucose) was noted in both groups as well [22].

The clinical effectiveness of telemedicine vs. a traditional care model was evaluated in 200 patients with uncontrolled T2D (HbA1c > 9%) who attended an outpatient diabetes clinic [23]. The telemedicine arm included patients that attended a virtual clinic between March and June 2020 and the traditional care model included patients who received in-person care between August and November 2020. The telemedicine group had a reduction of 1.82% ± 1.35% (95% Cl = 1.56–2.09, *p* < 0.001) when compared to the traditional care model, which had a mean reduction of 1.54% ± 1.56% (95% Cl = 1.23–1.85, *p* < 0.001 [23].

Another study explored the impact of telemedicine on HbA1c in high-risk patients (HbA1c > 8%) with T2D before and after the implementation of a pharmacist-led telehealth service [24]. The study evaluated the change in HbA1c between the pre-COVID-19 group (August 2019–February 2020) and the COVID-19 group (March 2020–October 2020). The study showed an HbA1c reduction of 1.3% in the pre-COVID-19 group vs. 2% in the COVID-19 group at three months follow-up, *p* = 0.305. An HbA1c reduction of 1.2% in the pre-COVID-19 vs. 2.2% in the COVID-19 group, *p* = 0.249 at six months follow-up, was also observed [24].

Finally, three retrospective studies enrolled both T1D and T2D patients to analyze the efficacy of telemedicine during the state of emergency [25,26,27]. Of these studies, one evaluated outpatient diabetes care and HbA1c levels during the 2020 pandemic to 2019 by comparing the 13 weeks before (pre-period) and after (post-period) the lockdown period (26 May–24 August 2020) with the same time frame in 2019 [25]. This study found a post-period HbA1c of 7.2% in 2020 and 7.2% in 2019 (*p* = 0.43) with a change in HbA1c of −0.1 and −0.2 from the pre-period, respectively (*p* < 0.001). A propensity analysis done between clinic visits vs. telemedicine visits in 2020 showed a reduction in HbA1c from baseline 7.6 to 7.5%, *p* = 0.023, with a difference reduction of –0.15 in the telemedicine compared with the clinic visit group that showed a reduction of HbA1c from 7.6 to 7.4%, *p* = 0.023 with a reduction of −0.23, *p* = 0.019 favoring clinic visit over telemedicine [26]. The second study conducted a multiple regression analysis of patients with T1D and T2D (N = 2727), which showed that following adjustment for sex and type of diabetes, lower pre-BMI, lower pre-HbA1c, younger age, and clinic visit and/or telemedicine visit were associated with a higher chance of achieving an HbA1c < 7% [26]. Lastly, a study conducted by Wong et al. analyzed a cohort of 504 patients with both T1D and T2D) [27]. The study assessed telehealth consultations that took place between 1st April 2020 and 1st September 2020 (Visit A) and compared it to the proportion of patients that attended a face-to-face encounter during the same months in the year 2019 (Visit B) and finally compared it to patients that attended the clinic between April and September 2020 and had been attending the clinic face-to-face for at least 12 months prior to the onset of the pandemic (Visit C). When assessing HbA1c available at all patients, the study found improvements in HbA1c of 7.8% ± 1.6% at Visit A when compared to 8.1 ± 1.4 at Visit B and 8.2 ± 1.7% at visit C (*p* < 0.001). Patients with T2D also had a lower HbA1c at visit A compared to visit B and visit C. However, in patients with T1D, there was no significant difference in glycemic control between visit A, visit B, and visit C, with an HbA1c of 8.3 ± 1.4%, 8.4 ± 1.7, and 8.4 ± 1.8, respectively [27].

### 3.2. Evidence from Prospective Studies

Three prospective studies evaluated the effect of telemedicine in improving glycemic control in individuals with T1D and T2D. Two of the three studies enrolled patients with T1D and one enrolled patient with T2D [28,29,30].

A pilot study, which included 166 patients with T1D, aimed to evaluate different glycemic outcomes collected during two virtual visits during the lockdown period [26]. The study considered different methods of insulin delivery and glucose monitoring for its assessment (CSII + CGM, MDI + CGM, and CSII or MDI + SMBG), showing that TIR increased from baseline to follow-up visits in all patients). There was a non-statistically significant improvement in TBR and GMI compared to baseline and statistically significant improvements in TAR and mean daily glucose (Table 2) [28]. Notably, the CSII and MDI+SMBG group displayed better improvements in the TAR from baseline compared to follow-up visits (40.0% ± 18.0% vs. 28.0% ± 15.0%, respectively; *p* = 0.03), a reduction in mean daily glucose (176± 49 mg/dL vs. 150 ± 25 mg/dL; *p* = 0.04), and improvement in GMI (7.5% ± 1.1% vs. 6.9% ± 0.6%; *p* = 0.04), and CV (36.0% ± 8.0% vs. 42.0%± 9.0%; *p* = 0.04) compared to the other groups. In a subgroup analysis, the authors found a significant improvement in TIR in those with a GMI > 7.5% as compared to those with a GMI < 7.5% [28].

Another study enrolled 87 patients with uncontrolled T1D diabetes (GMI > 9%) and followed patients between March and June 2020 through online visits, conferences, and group sessions [29]. The authors evaluated the number of hospitalizations for DKA and severe hypoglycemia causing loss of consciousness or seizures and, as a secondary endpoint, reduction in GMI. The participant’s outcomes were compared to patient data from patients with HbA1c > 9% in the TID exchange. The study found fewer hospitalizations for DKA in the enrolled patients vs. T1D exchange (2.2 vs. 6.71%), fewer episodes of severe hypoglycemia in telemedicine vs. T1D exchange (1.1% vs. 7%) and change in mean GMI of −0.66% (reduced from 9.91 to 9.25%) during this period [29].

Finally, a study assessed 130 T2D patients with HbA1c > 9% who attended a virtual integrated care clinic over four months during the pandemic. Using Hb1Ac as a marker for glycemic control, this single-arm observational study showed a decrease in pre-intervention HbA1c from 9.98 ± 1.33 to 8.32 ± 1.31 (*p* < 0.001) post-intervention [30]. 

## 4. Discussion

Hyperglycemia, hypoglycemia, or increased glucose variability have been associated with increased morbidity, frequent hospitalizations/emergency department (ED) visits, and higher mortality [31,32,33,34,35]. Achieving better glucose control is important and frequent clinic visits are often required for medication adjustments. In addition, many patients with diabetes have underlying comorbidities that restrict mobility or live in remote/rural areas posing barriers to seeking in-person care. Telemedicine can serve as an alternative method of providing less time consuming and more accessible patient care, it is just a matter of embracing the technological options already available [36]; by doing so, it could allow quicker titration of diabetes medications, improving monitoring and glycemic parameters compliance in medication taking, and improving outcomes [37]. Although telemedicine can be an option, most visits are still performed in-person. Telemedicine can utilize different telecommunication options, among them video conference applications, which have expanded following the COVID-19 declaration. With a growing number of patients using smartphones and having Internet access (more than 85% of the US population using smartphones [38] and 93% having Internet access [39], a figure that is constantly rising), utilizing the Internet to transfer data and perform telemedicine should not be considered a futuristic solution for healthcare delivery, but an option to use at the present time.

**Figure 2:**
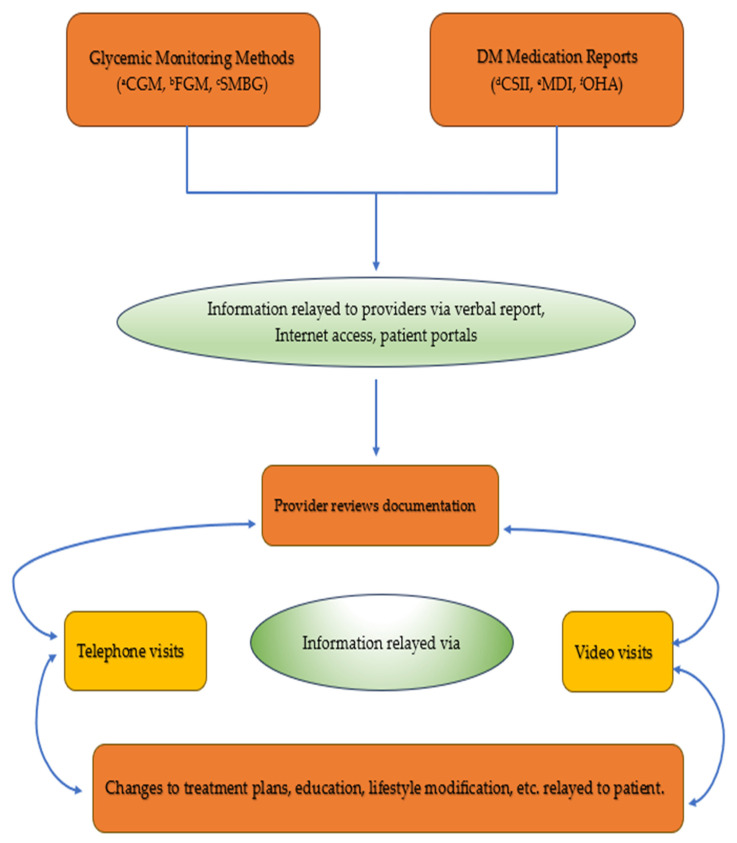
Use of telemedicine for remote glucose monitoring. ^a^ CGM: Continuous Glucose Monitor; ^b^ FGM: Flash Glucose Monitor; ^c^ SMBG: Self-Monitoring Blood Glucose; ^d^ CSII: Continuous Subcutaneous Insulin Infusion; ^e^ MDI: Multiple Daily Injection; ^f^ OHA: Oral Hypoglycemic Agent.

Overall, telemedicine proved to be a timely solution in the face of the COVID-19 outbreak, allowing for appropriate glycemic control (Figure 2). The studies reported were conducted across different countries worldwide, showcasing a diverse population. They focused on patients with both type 1 and type 2 diabetes with different treatment modalities (insulin pump, multiple daily insulin injections, oral hypoglycemic agents, GLP-1 agonists) and different glucose monitoring methods (CGM, FGM, SMBG) (Table 1 and Table 2). Notably, the retrospective studies focusing on individuals with T1D showed improvements across various glycemic control measures regardless of the treatment modality [18,19,20]. These studies showed improvements in TIR, reductions in TAR, improvements in mean glucose values, and reductions in HbA1c% and GMI [18,19,20]. In the retrospective studies following patients with T2D, most studies found that the use of telemedicine led to reductions in HbA1c [21,24], with one study showing it to be equally effective as the standard of care model [22]. Similar findings were observed in studies that used mixed population of patients with T1D and T2D, in which telemedicine led to improvements in HbA1c [25,26,27].

It is crucial to acknowledge that the heterogenicity of these studies, including variations in outcomes, patient population, sample size, methods of glycemic monitoring, and insulin delivery restricts the clear interpretation of telemedicine’s role on diabetes management. While some prospective studies showed improvements in TIR, mean glucose value, and reductions in TAR [18,19,20], it is important to note that their small sample size could contribute to their results. Additionally, while reductions in HbA1c were noted across all telemedicine groups [21,22,23,24,25,26,27,30], some studies only found slight improvements [21], and another found no statistical significance among the groups [24]. Furthermore, studies focused on T1D patients used CGM devices to monitor glycemic control [18,19,20], potentially confounding the role of telemedicine. Therefore, while the telemedicine groups did show improvements in glycemic control, the use of CGM devices could have contributed to their overall improvement. Nonetheless, the prospective study conducted by Parise et al. [28] highlighted that in all patients with T1D, the telemedicine group showed improvements in TIR regardless of the glucose monitoring method. In addition, lockdown could have allowed patients to have more time to allocate to diabetes care, hence confounding the effect of telemedicine. It should be also noted that that current evidence is based on retrospective-observational studies, as the number of prospective studies which were conducted evaluating the role of telemedicine in patients with diabetes during the COVID-19 era is much smaller. Large randomized clinical trials are needed to evaluate the role of telemedicine in glycemic control in patients with diabetes.

Even with the heterogenicity of these studies, telemedicine showed improvements in diabetes control, across different monitoring methods and treatment modalities, proving effective in diabetes management across various studies; however, even those with similar glycemic control outcomes did not exhibit a clear clink between telemedicine and specific measures. Furthermore, not all articles focused on the impact of hospitalization or events such as DKA or hypoglycemia. We also did not focus on the financial impact of telemedicine, as we deemed that that deserves a separate analysis of its own.

The use of diabetes technology, such as CGM or FGM, has emerged as an important tool for diabetes management. As shown in the studies presented, such technology seems to make diabetes management suitable for telemedicine by allowing a provider to review data remotely. With the development of new integrative information sharing, telemedicine can impact how diabetes is managed in the future. Remote monitoring can lead to improved glycemic measures, and as healthcare becomes more integrative, individuals with diabetes can be closely monitored by their physician. Expanding on these services will further allow those with diabetes to play a more active role in the management of their chronic illness. However, it faces significant hurdles such as cost, patient education, and need of technology training. As mentioned earlier, telemedicine was not widely used prior to the COVID-19 pandemic, but quickly became adopted as an instrument for diabetes care during the initial stages [17]. Our article emphasizes the variability in the current literature regarding the use of telemedicine in diabetes management, highlighting that while telemedicine has been shown to be a safe, valid, and adequate option for managing chronic diseases such as diabetes [17], its precise role is yet to be understood. Furthermore, as restrictions are lifted and life returns to normal, this article seeks to highlight the need for randomized clinical trials that assess telemedicine’s impact beyond the pandemic’s initial phases and how it can be optimized for diabetes management as we move forward from the pandemic.

## 5. Conclusions

Across different studies reported in this review, telemedicine was shown to be an effective tool for the management of diabetes, illustrating potential to be the new standard of care. Indeed, telemedicine became an invaluable tool during the initial phases of the pandemic and continues to prove crucial in managing chronic diseases. The evolution of technology is set to play a crucial role in future diabetes care. Tools such as continuous glucose monitors, insulin pumps, and smart pens not only have a positive impact on diabetes management but can also allow telemedicine to become standard practice in this group of patients. The heterogenicity and variability in the study results make it apparent that we do not yet fully understand how to best optimize telemedicine for the management of diabetes. Yet, these studies showed that telemedicine can be a promising and safe method of health care delivery in patients with diabetes compared to in-person visits.

## Figures and Tables

**Table 1:** Retrospective studies which examined telemedicine use in patients with DM.

Ref.	Country	Population	Study Aim	DM Regimen	Glycemic Monitor	Results
[18]	Italy	T1DM ^a^ n = 30	Evaluating the metrics of glycemic control in T1DM patients using HCL pumps across 4 different time points during lockdown (Time 0: pre-lockdown; Time 1: First 2 weeks of lockdown; Time 2: Last 2 weeks of lockdown; Time 3: post-lockdown).	CSII ^c^	CGM ^g^	Improvement in mean glucose value (155 mg/dL in Time 0 vs. 153 mg/dL in Time 3, *p* = 0.004). Improvement in TIR ^l^ (70–180 mg/dL) from 68.5% in Time 0 vs. 73.5% in Time 3, *p* = 0.012. Reduction in TAR ^m^ level 2 (251–400 mg/dL) from 6% in Time 0 vs. 4% in Time 3, *p* = 0.002). No difference in level 1 (54–69 mg/dL) or level 2 (<54 mg/dL) hypoglycemia (1% in Time 0 through Time 3 *p* = 0.190 vs. 0% in Time 1 through Time 3, *p* = 0.183 respectively) Increase in time spent in auto-mode (91.5% in Time 0 vs. 94% in Time 3, *p* = 0.018).
[19]	Italy	T1 DM ^a^ n = 71	Analyze data from CGM ^g^ or FGM ^h^ systems during the lockdown diabetes and compare data obtained 4 weeks before and 4 weeks after structured telephone visits.	MDI ^d^ and CSII ^c^	CGM ^g^ FGM ^h^	Reduction in GMI ^o^ from 7.16 to 7.05 *p* = 0.002 Reduction in mean glucose value from 161.1 mg/dl to 156.3 mg/dL, *p* = 0.001. Increase in TIR ^l^ (70–180 mg/dL) from 63.6% to 66.4%, *p* < 0.001. Reduction of the time TAR ^m^ (>180 mg/dL) from 33.4% to 30.5%, *p* = 0.002. No change in time in TBR ^n^ (<70 mg/dL) with 3% vs. 3.2% *p* = 0.6 Among those that were managed with MDI ^d^, there was an improvement in average glucose values (mean glucose 163.7 vs. 159.74 in those using MDI ^d^ 4 weeks before and 4 weeks after TM ^q^ visit respectively, *p* = 0.05). Among those managed with CSII ^c^, there was also an improvement (mean glucose of 157.9 vs. 152.6 in those using CSII ^c^ 4 weeks before and 4 weeks after TM ^q^ visit, respectively, *p* = 0.003).
[20]	Saudi Arabia	T1 DM ^a^ n = 101	Assess glycemic control in those who attended a telemedicine visit during the 6-week lockdown period vs. those who did not.	MDI ^d^ and CSII ^c^	FGM ^h^	Improvement in average blood glucose from 180 to 159 mg/dL in those who attended a telemedicine visit, *p* < 0.01 vs. 159.5 to 160 mg/dL, *p* = 0.99 in those who did not have a telemedicine visit. Improvement in TIR ^l^ (70–180 mg/dL) from 46 to 55% in those who attended a telemedicine visit, *p* < 0.01 vs. 58 to 57%, *p* = 0.20 in those who did not have a telemedicine visit. Improvement in TAR ^m^ (>180 mg/dL) from 48 to 35%, *p* < 0.01 vs. 35% to 35%, *p* = 0.83 in those who did not have a telemedicine visit. Improvement in GMI ^o^ from 7.7 to 7.2%, *p* = 0.03 vs. 7.3 to 7.2%, *p* = 0.65 in those who did not attend a telemedicine visit. There was no significant change in TBR ^n^ (<70 mg/dL) with 3 to 5%, *p* = 0.06 in those who had a telemedicine visit vs. 4.5 to 5.5%, *p* = 0.40 in those who did attend a telemedicine visit. No significant changes in hypoglycemic events from 6 to 8 events *p* = 0.22 vs. 11 to 8 events, *p* = 0.28 in those who attended a telemedicine visit vs. those who did not, respectively.
[21]	Italy	T2DM ^b^ n = 269	Difference in Hb A1c and body weight between May–June 2020 (after lockdown) vs. November–February 2020 (before lockdown).	Insulin OHA ^e^ GLP-1 ^f^	N/A ^t^	HbA1c ^p^ 7.3% ± 3.1% pre-lockdown vs. 7.2% ± 3.2%, post lockdown (*p* < 0.01), respectively. Weight 83.2 ± 16.8 kg vs. 81.6 ± 16.4, *p* < 0.01 pre and post lockdown, respectively.
[22]	India	T2DM ^b^ n = 96	Compare glycemic control in those evaluated through telemedicine vs. those evaluated in person (IPV) ^r^	MDI ^d^, OHA ^e^, GLP-1 ^f^	FPG ^i^ PPPG ^j^	Improvement in baseline HbA1c ^p^ from baseline 8.7% ± 1.8% to 6.9% ± 1.1% in the telemedicine compared to a reduction in HbA1c ^p^ from baseline 8.6% ± 2.1% to 7% ± 1%, *p* = 0.88 in the IPV ^r^ group. Improvement in baseline FPG ^i^ from baseline 184.1 ± 69 to 120.3 ± 20.8 in the telemedicine group vs. improvement from baseline 184.9 ± 73.1 to 118.6 mg/dL, *p* = 0.761 in the IPV ^r^ group. Improvement in baseline PPPG ^j^ from baseline 244.2 ± 70.2 to 155.1 ± 30.3 in the telemedicine group vs. improvement from baseline 289.8±112.3 to 155.1 vs. 172.3 mg/dL, *p* = 0.104 in the IPV ^r^ group.
[23]	Saudi Arabia	T2DM ^b^ n = 200	Impact of telemedicine on glycemic control on patients with uncontrolled diabetes (HbA1c > 9%) vs. traditional care mode between March and June 2020.	Insulin	SMBG ^k^	Reduction in HbA1c ^p^ in the telemedicine group vs. the traditional care group from 10.31% to 8.49% vs. 10.53 to 8.99%, *p* < 0.001.
[24]	United States	T2DM ^b^ n = 91	Determine the overall change in A1C between August 2019 and February 2020 (pre- COVID-19 group) and March and October 2020 (COVID-19 group) in a pharmacy driven telehealth services during the COVID-19 public health emergency (PHE) in patients with HbA1c ^p^ > 8%.	N/A ^t^	N/A ^t^	After 3 months follow up, there was a reduction in HbA1c ^p^ of 1.3% in the pre COVID-19 group vs. 2% reduction in the COVID-19 group, *p* = 0.305. After 6 months follow up, there was a reduction in HbA1c ^p^ of 1.2% in the pre COVID-19 vs. 2.2% in the during COVID-19 group, *p* = 0.249.
[25]	Japan	T1DM ^a^ = 407 T2DM ^b^ = 6534 n = 6941	Compare outpatient diabetes care and HbA1c ^p^ levels during the COVID-19 pandemic in 2020 with 2019.	MDI ^d^, OHA ^e^, GLP-1 ^f^	N/A ^t^	Propensity analysis done between clinic visit vs. telemedicine visits in 2020 showed a reduction in HbA1c ^p^ from baseline 7.6 to 7.5%, *p* = 0023, with a difference reduction of –0.15 in the telemedicine compared with the clinic visit group that showed a reduction of HbA1c ^p^ from 7.6 to 7.4%, *p* = 0.023 with a reduction of −0.23, *p* = 0.019 favoring clinic visit over telemedicine.
[26]	Japan	T1DM ^a^ = 171 T2DM ^b^ = 2556 n = 2727	Assess the impact of telemedicine on HbA1c ^p^ between the pre-emergency period (February–April 2020) and the post-emergency period (May–July 2020).	N/A ^s^	N/A ^t^	Following adjustment for sex and type of diabetes lower pre-BMI ^s^, lower pre-HbA1c ^p^, younger age and clinic visit and/or telemedicine visit, was associated with higher chance of achieving a HbA1c ^p^ < 7%.
[27]	Australia	T1DM ^a^ = 92 T2DM ^b^ = 412 n = 504	Assess attendance rate, glycemic control, and unplanned hospital admissions between April 1st 2020 and September 2020 (visit A) compared to patients in the same time period in 2019 (visit B) and compared to patients that attended the clinic between April and September 2020 and had been attending the clinic for at least 12 months prior to the onset of the pandemic (Visit C).	MDI ^d^	SBMG ^k^	Improvement in HbA1c ^p^ when compared to 8.1 ± 1.4 at Visit B and 8.2 ± 1.7% at visit C (*p* < 0.001). Patients with T2DM had a lower HbA1c ^p^ at visit A (7.8 ± 1.4%) compared to visit B (8.0 ± 1.6, *p* < 0.5) and visit C (8.2 ± 1.7, *p* < 0.001). No difference in unplanned admissions to hospital among cohort between April and September 2020 (n = 58; 9.2%) compared with those in the same period in 2019 (n = 75; 11.9%; *p* = 0.100).

^a^ T1DM: type 1 diabetes; ^b^ T2DM: type 2 diabetes; ^c^ CSII: continuous subcutaneous insulin infusion; ^d^ MDI: multiple daily injection; ^e^ OHA: oral hypoglycemic agent; ^f^ GLP-1 RA: glucagon-like peptide-1 receptor agonists; ^g^ CGM: continuous glucose monitor; ^h^ FGM: flash glucose monitor; ^i^ FPG: fasting plasma glucose; ^j^ PPPG: post prandial plasma glucose; ^k^ SMBG: self-monitoring blood glucose; ^l^ TIR: time in range; ^m^ TAR: time above range; ^n^ TBR: time below range; ^o^ GMI: glucose management indicator; ^p^ HbA1c: hemoglobin A1c; ^q^ TM: telemedicine; ^r^ IPV; ^s^ BMI: body mass index; ^t^ N/A: not applicable.

**Table 2:** Prospective studies which examined telemedicine use in patients with DM.

Ref.	Country	Population	Study Aim	DM Regimen	Glycemic Monitor	Results
[28]	Italy	T1DM ^a^ n = 166	Assess glycemic control at baseline vs. follow up on patients that completed 2 virtual visits between 10 March 2020 and 3 June 2020.	MDI ^d^ CSII ^c^	CGM ^g^ SMBG ^h^	Increase in TIR ^i^ (70–180 mg/dL) in all patients from baseline to follow up visit (62% ± 18% vs. 65% ± 16%, *p* = 0.02, *p* = 0.02) Improvement in TBR ^k^ (<70 mg/dL) from 3.5% vs. 3.4%, *p* = 0.58, improvement in TAR ^j^ (>180 mg/dL) from 34% vs. 32%, *p* = 0.08, mean daily glucose from 163 mg/dL vs. 153 mg/dL, *p* = 0.25 and GMI ^l^ from 7.2% vs. 7.1%, *p* = 0.23 from baseline to follow up respectively in all patients. Greater improvement in TIR ^i^ (70–180 mg/dL) in those with GMI ^l^ > 7.5% (45.0% ± 15.0% vs. 53.0% ± 18.0%, *p* < 0.01) compared to those with GMI ^l^ < 7.5% (68.0% ± 15% vs. 69.0% ± 15%, *p* = 0.98). No hospitalization or ED visits for DKA ^n^ or hypoglycemia.
[29]	United States	T1DM ^a^ n = 87	Impact of telemedicine on the number of hospitalizations for DKA ^n^, incidence of severe hypoglycemia and GMI ^l^ among patients with GMI ^l^ > 9%s in a 3-month study compared with patient with uncontrolled DM in the T1D Exchange with HbA1c ^m^ > 9%.	MDI ^d^ CSII ^c^	CGM ^g^	Hospitalizations for DKA ^n^ were 2.2% in the telemedicine group vs. 6.71% in the T1D Exchange. Fewer episodes of severe hypoglycemia in telemedicine vs. T1D exchange (1.1 vs. 7%). Among those that have been followed by telemedicine, there was a change in mean GMI ^l^ of −0.66% (from 9.91 to 9.25%).
[30]	Saudi Arabia	T2DM ^b^ n = 130	Impact of telemedicine on glycemic control on high-risk patients (HbA1c ^m^ > 9%) attending a virtual integrated care clinic over a 4-month period during the pandemic	MDI ^d^ OHA ^e^ GLP-1 ^f^	SMBG ^h^	The HbA1c ^m^ decreased from 9.98 ± 1.33 pre-intervention to 8.32 ± 1.31 post-intervention, *p* < 0.001.

^a^ T1DM: Type 1 diabetes; ^b^ T2DM: Type 2 diabetes; ^c^ CSII: Continuous subcutaneous insulin infusion; ^d^ MDI: Multiple daily injection; ^e^ OHA: oral hypoglycemic agent; ^f^ GLP-1 RA: Glucagon-like peptide-1 receptor agonists; ^g^ CGM: Continuous Glucose Monitor; ^h^ SMBG: Self-Monitoring Blood Glucose; ^i^ TIR: Time in Range; ^j^ TAR: Time Above Range; ^k^ TBR: Time below range; ^l^ GMI: Glucose Management Indicator; ^m^ HbA1c%: Hemoglobin A1c; ^n^ DKA: diabetes ketoacidosis.
